# Replicating Strand Asymmetry in Bacterial and Eukaryotic Genomes

**DOI:** 10.2174/138920212799034794

**Published:** 2012-03

**Authors:** Feng-Biao Guo

It is my pleasure as a Guest Editor of Current Genomics to present you with a ‘hot topic issue’ on DNA replication.

DNA replication adopts a set of asymmetric mechanisms. One of them is the division of leading and lagging strands. In 1991, the nucleotide composition bias between the two replicating strands was originally found in genomes of echinoderm and vertebrate mitochondria. In the following twenty years, more and more bacterial genomes are found to have much different nucleotide composition between the two replicating strands. More importantly, eukaryotes and even mammalians are found to have such strand bias (asymmetry) in recent years. Besides composition bias, there are also other types of biases between two replicating strands, such as gene orientation bias, gene function bias and substitution rate bias. This topic of replicating strand bias has attracted numerous researchers to perform abundant and in-depth researches. In my view, it will continue to be one of the hot topics of genomics. A better understanding of replicating strand asymmetry will greatly advance our knowledge about the mechanism of DNA replication.

This theme issue aims mainly to summarize various types of biases between the two replicating strands in bacterial and eukaryotic genomes. Another aim is to try to elucidate the underlying mechanisms of various strand biases. Therefore, this theme issue includes papers reviewing types, extents, application and underlying mechanism of strand asymmetry both in bacterial and eukaryotic genomes.

K. Arakawa and M. Tomita reviewed the measures of strand bias that have been proposed to date, including the ΔGC skew, the predictability score of linear discriminant analysis for gene orientation and so on. These methods measure the general composition bias, gene distribution bias and the composition bias of certain oligonucleotides, respectively. Although these measures were predominantly designed for and applied in analyzing replication-related mutational processes of prokaryotes, the authors also give research examples in eukaryotes.

X. Xia summarized the diverse patterns of strand asymmetry among different taxonomic groups and made four suggestions. The survey was involved with bacterial, archaeal and mitochondrial genomes. The four suggestions concern the numbers of replication origins and replicating mechanism in certain taxa.

Lin *et al.* reviewed composition bias between light and heavy strands of animal mitochondrial genomes. They discussed the influence of replication-associated mutation pressure on nucleotide and amino acid compositions as well as gene organization in these genomes.

H. Seligmann reviewed coding constraints on replication-associated strand composition bias in mitochondrial genomes. He concludes that the bias is the most at 3rd and least at 2nd codon positions across mitochondrial genomes. There are not bias at synonymous positions forming off frame stops and in regions coding for putative overlapping genes. Also, he compared transcription and replication associated biases of nucleotide composition and mutation rate.

The review from V.V. Khrustalev and E.V. Barkovsky focuses on their hypothesis on the formation of replicating strand asymmetries in prokaryotes. In this review, they summarized various proofs supporting the theory. The hypothesis is that the combined effects of cytosine deamination and guanine oxidation produce more nonsense mutations in genes from lagging strands than in genes from leading strands, and hence lead to the composition bias and gene density bias.

All of the above papers reviewed mainly on computational researches. Because all types of strand asymmetries derive from the asymmetric mechanism of replication and/or transcription, we have also arrange one review concerning experiment works of DNA replication and transcription. In the paper by Lin and Pasero, mechanisms of the two processes are touched and the focus is put on interference between them.

Overall, these reviews provide a fascinating survey of the types, extents, mechanisms and applications of replicating strand asymmetry. I hope that this issue will be useful for the researchers and students in the filed. Finally, I would like to thank all the reviewers for their valuable suggestions and comments, which have led to the improvement of review articles.

